# Clinically Available Low Intensity Ultrasound Devices do not Promote Axonal Regeneration After Peripheral Nerve Surgery—A Preclinical Investigation of an FDA-Approved Device

**DOI:** 10.3389/fneur.2018.01057

**Published:** 2018-12-04

**Authors:** Simeon C. Daeschler, Leila Harhaus, Konstantin D. Bergmeister, Arne Boecker, Bernd Hoener, Ulrich Kneser, Philipp Schoenle

**Affiliations:** ^1^Department of Hand, Plastic and Reconstructive Surgery, Burn Center, University of Heidelberg, BG Trauma Hospital Ludwigshafen, Ludwigshafen, Germany; ^2^Faculty of Social Sciences and Law, SRH University Heidelberg, Heidelberg, Germany

**Keywords:** experimental studies, microsurgery, peripheral nerve injuries, nerve surgery, nerve regeneration, reinnervation, ultrasound therapy

## Abstract

The slow axonal regeneration and consecutive delayed muscle reinnervation cause persistent functional deficits following peripheral nerve injury, even following sufficient surgical nerve reconstruction. Preclinically, adjunct ultrasound therapy has shown to significantly accelerate nerve regeneration and thereby improve muscle function compared to nerve reconstruction alone. However, although FDA-approved and clinically well-tested ultrasound devices for other conditions such as delayed-healing fractures are available, they have not been investigated for peripheral nerve injury yet. Aiming to provide a fast clinical translation, we evaluated EXOGEN (Bioventus LLC, Durham, USA), a clinical device for low-intensity ultrasound therapy in various treatment intervals following peripheral nerve surgery. Sixty rats, randomized to five groups of twelve animals each, underwent median nerve transection and primary epineural nerve reconstruction. Post-surgically the ultrasound therapy (duration: 2 min, frequency: 1.5 MHz, pulsed SATA-intensity: 30 mW/cm^2^, repetition-rate: 1.0 kHz, duty-cycle: 20%) was applied either weekly, 3 times a week or daily. A daily sham-therapy and a control-group served as references. Functional muscle testing, electrodiagnostics and histological analyses were used to evaluate nerve regeneration. The post-surgically absent grip strength recovered in all groups and increased from week four on without any significant differences among groups. The weekly treated animals showed significantly reduced target muscle atrophy compared to sham-treated animals (*p* = 0.042), however, with no significant differences to three-times-a-week-, daily treated and control animals. The number of myelinated axons distal to the lesion site increased significantly in all groups (*p* < 0.001) without significant difference among groups (*p* > 0.05). A full recovery of distal latency was achieved in all groups and muscle function and CMAP recurred with insignificant differences among groups. In conclusion, the clinically available FDA-approved ultrasound device did not promote the axonal regeneration following nerve injury in comparison to control and sham groups. This is in contrast to a conclusive preclinical evidence base and likely due to the insufficient ultrasound-intensity of 30 mW/cm^2^. We recommend the clinical investigation of 200–300 mW/cm^2^.

## Introduction

Traumatic peripheral nerve injuries predominantly affect young and previously healthy patients in their occupational key years, thus leading to a large socioeconomic and personal burden (1, 2). Despite an improved understanding of the pathophysiology, the slow axonal regeneration remains an unsolved key issue, limiting the functional outcome following nerve surgery (3–5). Severe axonal trauma causes Wallerian degeneration of the distal nerve segment and ideally launches the subsequent axonal regeneration. To provide structural guidance to the re-growing axons, nerve injuries are microsurgically reconstructed either by primary repair or, if a tension-free coaptation is impossible, by autologous nerve grafts as the gold standard or by nerve conduits. However, even following sufficient reconstruction, the limited axonal regeneration in combination with frequently long regeneration distances lead to prolonged muscle denervation. Consequently, target muscle fibers undergo fibrotic change and become progressively less receptive to regenerating axons, defining a limited time-frame for functional muscle reinnervation (5, 6). Here, adjunct therapies to accelerate the axonal growth process following reconstructive nerve surgery could provide substantial clinical benefits. However, these therapies have not been explored clinically. Experimentally, the stimulation of physiological processes that occur following nerve injury represents a promising approach to improve the axonal regeneration. Following nerve injury, Schwann cells are known to play a key role in axonal degeneration and regeneration. They are involved in phagocytosis of the cellular debris following axonal breakdown and provide a pivotal structural guidance and trophic support to the regenerating axons (7, 8). Repetitive application of ultrasound therapy following nerve surgery has been shown to significantly enhance these supportive effects of Schwann cells and thereby improve the functional outcome (9–13). In a recent meta-analysis we found low-intensity ultrasound to positively affect motor function recovery, nerve conduction velocity, compound muscle action potential as well as distal axon number and –myelination following nerve injury. Given the conclusive preclinical evidence base in the absence of negative side effects, the clinical investigation of this promising approach represents the next step in a bench-to-bedside translation. In other disciplines the stimulation of tissue regeneration via adjunct low intensity ultrasound therapy is already part of the clinical routine. Ultrasound devices for conditions such as acute and delayed healing fractures are successfully applied clinically and have been shown to be cost-effective, universally available and easy to use with excellent compliance rates of around 90% (14). Furthermore, a transducer-integrated software allows easy clinical supervision of outpatient therapies. Given these positive clinical experiences and the superior recovery of low intensity ultrasound treated nerves, the investigation of such available market-approved devices following nerve surgery would provide a fast clinical translation. In this study, we evaluated an FDA-approved low-intensity ultrasound device named EXOGEN (Bioventus LLC, Durham, USA) as adjunct treatment following neurotmetic median nerve lesion and primary repair. This forelimb injury model with the gold standard primary nerve reconstruction represents a frequent clinical situation and thereby aims to further facilitate the transferability of our results into clinical practice (1, 15, 16).

## Materials and Methods

The study is reported according to the ARRIVE guidelines (17).

### Ethical Statement and Experimental Animals

The experimental protocol (file reference 23 177-07/G 15-7-048) was approved by the German governmental authorities (Landesuntersuchungsamt Koblenz, Rhineland Palatinate) on the 3rd July 2015 according to the German animal welfare act. We included 60 female Sprague Dawley rats (age 6–8 weeks, initial body weight 180–200 g) in this study. For animal housing, standard Macrolon cages type four with fresh water and pellet food *ad libitum* were provided, according to the EU-directive 2010/63/EU (18). Room temperature around 22°C and a constant circadian rhythm of 12/24 h illumination were automatically maintained.

### Study Design

The experimental animals (*n* = 60) were randomly allocated into five study groups of 12 animals each, using a permuted block randomization procedure. The allocation was concealed for the microsurgeon and experimenter, being only accessible for the ultrasound therapist. All experimental groups underwent identical initial neurotmetic nerve injury and subsequent reconstruction procedure but received different post-surgical ultrasound therapy regimen: One group received EXOGEN ultrasound therapy once a week, a second group 3 times a week and a third group received daily EXOGEN ultrasound therapy. As a reference, a sham-group got daily sham therapy with the transducer switched-off and the control group received no post-surgical treatment (Table 1).

**Table 1:** Study design; This table presents the design of the experimental *in-vivo* study including sample size, treatment regimen and time points of outcome testing.

**Groups**	***n***	**Treatment regimen**	**Outcome testing for all groups**		
			**Pre-surgery**	**Week one to -seven post-surgery**	**Week eight post-surgery**
Weekly	12	US 1x/week	Grasping test	Weekly grasping test	•Grasping test •Electrodiagnostics (CMAP, distal latency) •Muscle wet weight •Nerve harvesting for histological analyses (axon number, axon distribution density)
Three times a week	12	US 3x/week		
Daily	12	US daily		
Sham	12	Sham-treatment daily		
Control	12	None		

*n, Sample size; US, Ultrasound; CMAP, Compound muscle action potential*.

### Experimental Procedure

In all experimental animals, the right median nerve was transected via micro scissor in the medial third of the upper arm, 5 mm proximal to its entrance in the cubital fossa. The nerve was immediately reconstructed using three epineural nerve sutures (10-0, non-absorbable, monofilament suture, Ethilon, Ethicon Germany by Johnson & Johnson Medical GmbH, Norderstedt, Germany). The wound was closed via subcutaneous and transcutaneous sutures (4-0 monofilament, absorbable suture, Monocryl, Ethicon Germany by Johnson & Johnson Medical GmbH, Norderstedt, Germany). A volatile oxygen-anesthetics mixture (95% oxygen and 5% Isoflurane) provided sufficient anesthesia and subcutaneous buprenorphine injections (0.05 mg/kg bodyweight) ensured adequate peri- and postoperative analgesia. For the post-surgical ultrasound therapy, the FDA-approved EXOGEN bone-healing device (Bioventus LLC, Durham, USA) was applied directly on the shaved skin above the nerve suture site, using standard coupling gel and with the following transducer output parameters: application time 2 min, frequency 1.5 ± 5% MHz, intensity 30 ± 30% mW/cm^2^ (SATA), repetition rate: 1.0 ± 10% kHz, Duty cycle: 20%. A short-term Isoflurane anesthesia prevented limb motion-induced ultrasound transmission artifacts.

### Primary Experimental Outcome

#### Motor Function Recovery

**Figure 1:**
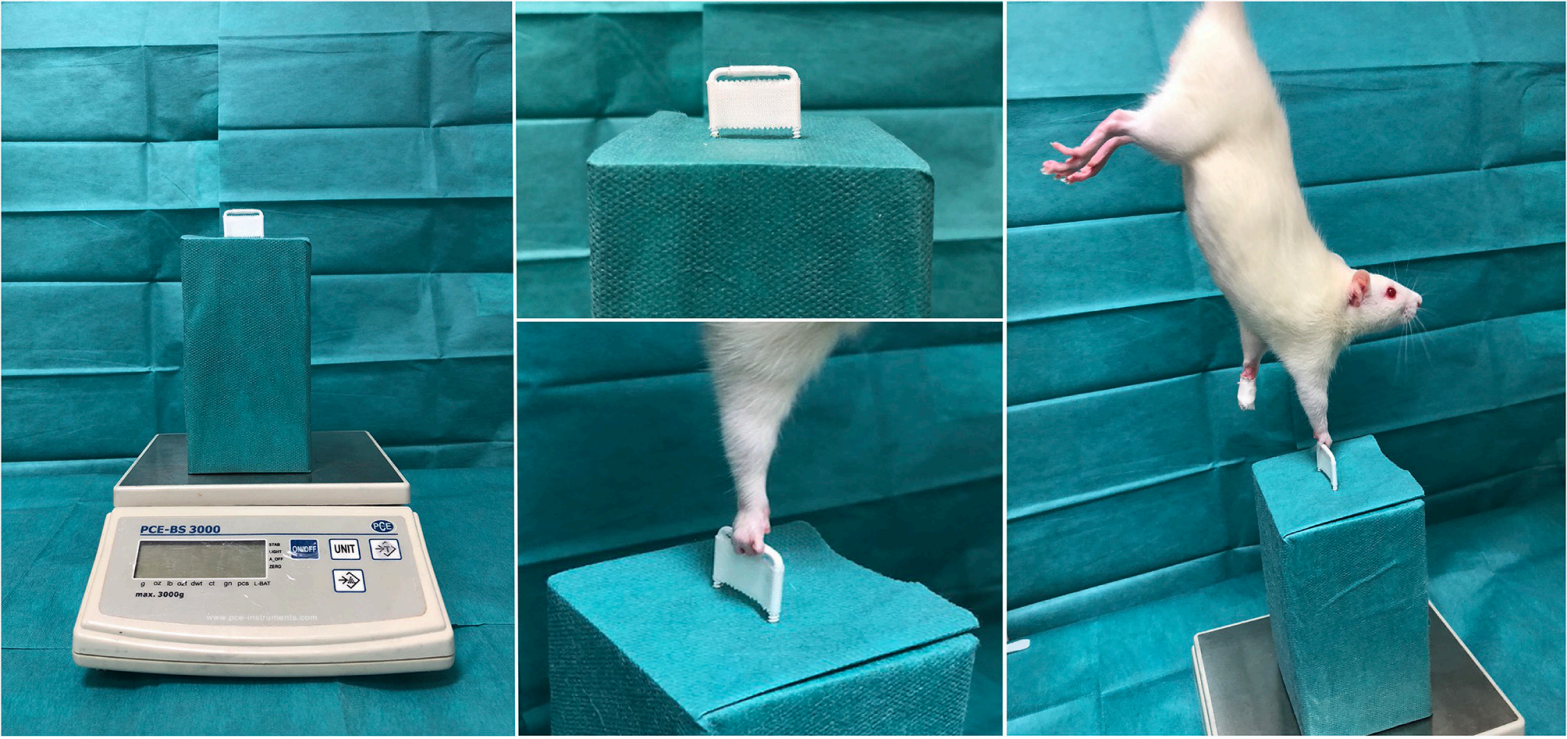
Grip strength test. Shown is the custom-made grip strength apparatus, coupled with a negative measuring scale. The white bar is designed to allow only the fingers to encompass the narrow grip-gap. Once the grip reflex is induced, the experimenter pulls the rat in a steady and reproducible upward motion, whilst the apparatus-coupled scale measures the maximum grip strength.

The finger flexors in rats are known to be exclusively innervated by the median nerve and are therefore suitable to evaluate the muscle function recovery via grip strength analyses (19). We applied a grasping test procedure to non-invasively detect the recurring grip strength of the reinnervated finger flexors following median nerve reconstruction (20). As proposed by Papalia and co-workers, a scale-coupled metal grip was suchlike customized, that only the fingers but not the whole paw were able to encompass the grip (21). This ensured the median nerve innervated finger flexors, but not the ulnar innervated wrist flexors, to contribute to the functional analysis. The contralateral forepaw was tape-immobilized and the animal was cautiously held on the base of its tail and carried above the apparatus to induce the grip reflex with the operated limb Figure 1. Once the animal grasped the grip, the blinded investigator pulled the animal in a steady and reproducible upward motion, whilst the apparatus-coupled scale measured the maximum grip strength. The highest value out of three repetitions was considered.

### Secondary Experimental Outcomes

Based on previous reports, electrodiagnostic and histologic analyses as well as denervation induced target muscle atrophy were further outcomes of interest to evaluate nerve regeneration and thereby treatment efficacy (22, 23).

#### Electrodiagnostics

The compound target muscle action potential (CMAP) and distal latency over the lesion site were analyzed 8 weeks post-surgery using an FDA-approved clinical EMG-device (NerveMonitor C2, Inomed Medizintechnik GmbH, Emmendingen, Germany). The nerve was carefully exposed in general anesthesia using minimal touch technique and the ulnar and the radial nerve were thoroughly dissected to avoid interfering electric signals. Via micromanipulator (Micromanipulator RH, Harvard Apparatus, Cambridge, Massachusetts, USA), a bipolar stimulating electrode (Mikro-Gabelsonde, Inomed Medizintechnik GmbH, Emmendingen, Germany) was gently placed 3 mm proximal to the suture site on the surface of the median nerve. The recording needle electrode (SDN-Elektrode, Inomed Medizintechnik GmbH, Emmendingen, Germany) was pricked in the flexor digitorum superficialis muscle (FDS) and a flexible latex-spacer defined a constant 22 mm distance between stimulating- and recording electrode. A monopolar, reference needle electrode (SDN-Elektrode, Inomed Medizintechnik GmbH, Emmendingen, Germany) was subcutaneously placed in the ipsilateral wrist. Isosmotic sodium chloride solution gently moisturized the nerve and avoided tissue dehydration.

**Figure 2:**
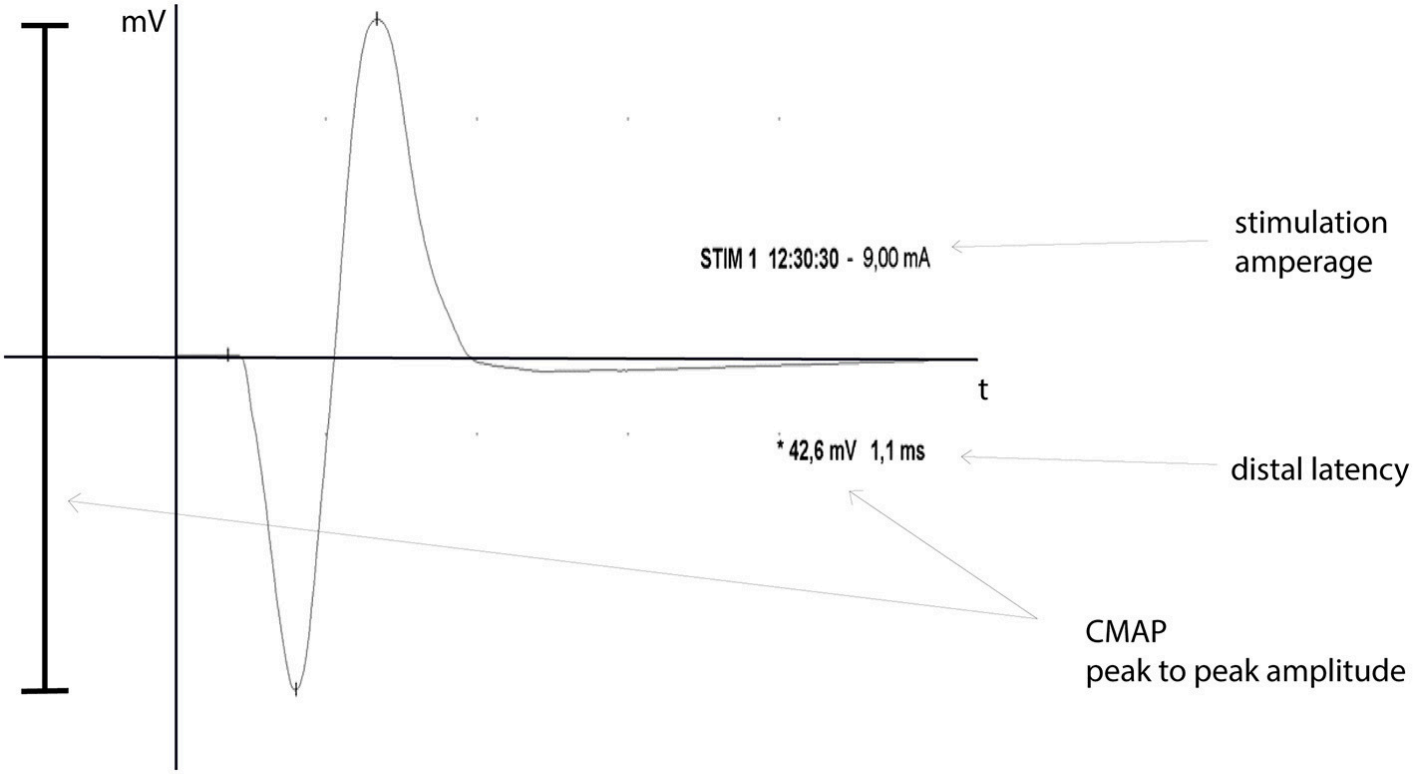
CMAP graph. Shown is an exemplary CMAP-graph, including nerve stimulation time and -amperage (STIM), peak-to-peak CMAP amplitude in millivolt (^*^) and the distal latency in milliseconds, as a graphic EMG-data output.

The maximum CMAP and distal latency were assessed via supra-maximal electric nerve stimulation proximal to the lesion site and the electric response was recorded from the FDS-muscle. The maximum CMAP peak-to-peak amplitude was electronically recorded in millivolt using clinical the EMG-device and corresponding software (EMG-Software, Version 3.0, NerveMonitor Software, Inomed Medizintechnik GmbH, Emmendingen, Germany). The time between supra-maximal stimulus and the first deviation from the baseline of the corresponding CMAP was measured as distal latency (see Figure 2).

#### Target Muscle Wet Weight

The ipsilateral FDS was harvested and weighted on a micro-scale (Adventurer pro, AV264CM, Ohaus corporation, Parsippany, USA) to quantify denervation induced target muscle atrophy. The muscle wet weight was normalized to the body weight.

#### Histological Analyses

**Figure 3:**
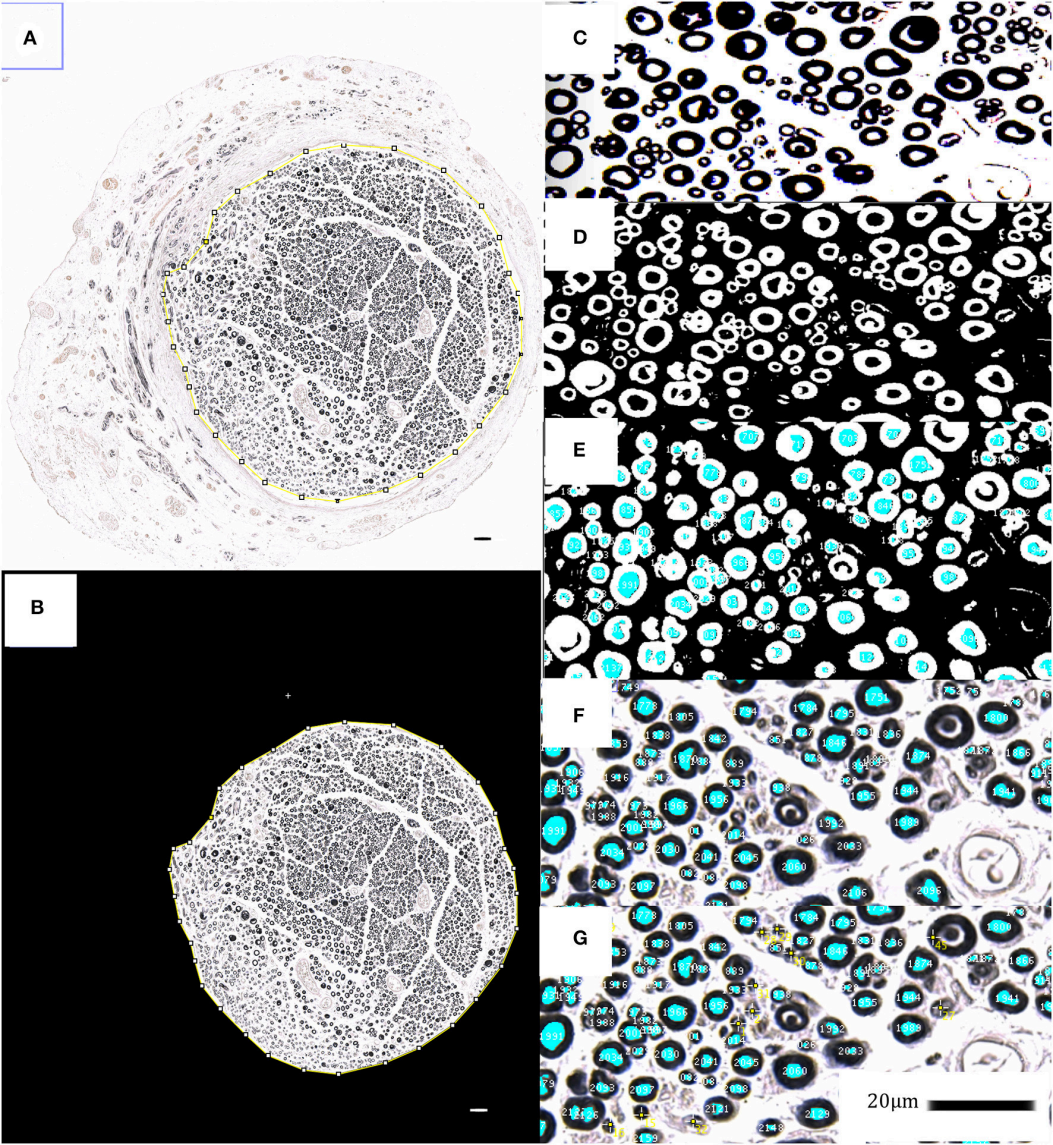
Histomorphometrical analyses; **(A–G)** show the applied semi-automatic algorithm via ImageJ for myelinated axon- and fascicle area detection and measurement in osmium tetroxide stained, paraffin embedded nerve cross sections. The fascicle area is isolated and analyzed in order to identify, tag and simultaneously count the black-stained myelin sheaths via synopsis of color thresholds, particle size and –circularity. The identified myelinated axons are displayed as an overlay-mask, which has been carefully checked for its sensitivity and specificity regarding axon identification. Untagged axons were manually selected and added to the overlay (yellow tags). 20 μm scale bars are included.

Based on previous work, showing a significant dose-dependent increase of myelinated axons per area, which significantly correlated with improved functional recovery, we analyzed the myelinated axon number and density to judge the EXOGEN treatment effects on regenerating nerves (23). For histological nerve examination, the reconstructed median nerve was harvested, immersion fixed in paraformaldehyde, stained in 2% osmium-tetroxide for 2 h, dehydrated in ascending alcohol series and embedded in paraffin blocks. The 2 μm microtome cross sections were cut between 8 and 6 mm distal to the nerve suture site and digitalized using the microscope Axio Imager M2 with the AxioCam MR (Carl Zeiss Microscopy GmbH, Jena, Germany) and corresponding software (AxioVision 4.8.2, Carl Zeiss Microscopy GmbH, Jena, Germany). For image processing and histomorphometric measurements, the scientific open source software ImageJ (Rasband, W.S., ImageJ, version 1.50 26th March 2016, U. S. National Institutes of Health, Bethesda, Maryland, USA) was used. Because heretofore no macro for osmium-stained, paraffin-embedded microtome nerve cross-sections was available, a custom-made algorithm was developed based on well-established methods for ultra-thin cross sections as introduced by Mackinnon and Hunter (24). This algorithm provided a semi-automatic detection and measurement of the fascicle cross sectional area, myelinated axon count as well as axon distribution density and was previously validated in 10 random nerve cross-sections, by matching the results with manual axon counts and measurements of a blinded experimenter (see Figure 3).

### Statistical Analysis

Statistical analyses were conducted using SPSS Statistics Version 24 by IBM. For descriptive statistics, the mean, the median and the standard deviation were calculated for each experimental group. Further statistical comparison between groups was conducted using one-way analysis of variance (ANOVA) in combination with a *post hoc* test with Bonferroni correction. To verify the necessary homogeneity of variances across the analyzed samples the Levene-test was previously performed. To evaluate correlation of experimental outcomes, the Pearson's-correlation coefficient was calculated. The chosen alpha level for all tests was 0.05 and thus *p* < 0.05 was considered as statistically significant.

## Results

Overall, 58 rats completed the experimental protocol and were included into the final analysis. Two animals (*n* = 1 of the weekly treatment group, *n* = 1 of control-group) had to be excluded due to non-treatment related issues (anatomical variation of nerve anatomy, anesthesia-related complication). The regular ultrasound treatments were successfully conducted in all included animals, with no complications or side effects. No adverse events were observed and especially no dermatological side effects occurred.

### Motor Function Recovery

**Figure 4:**
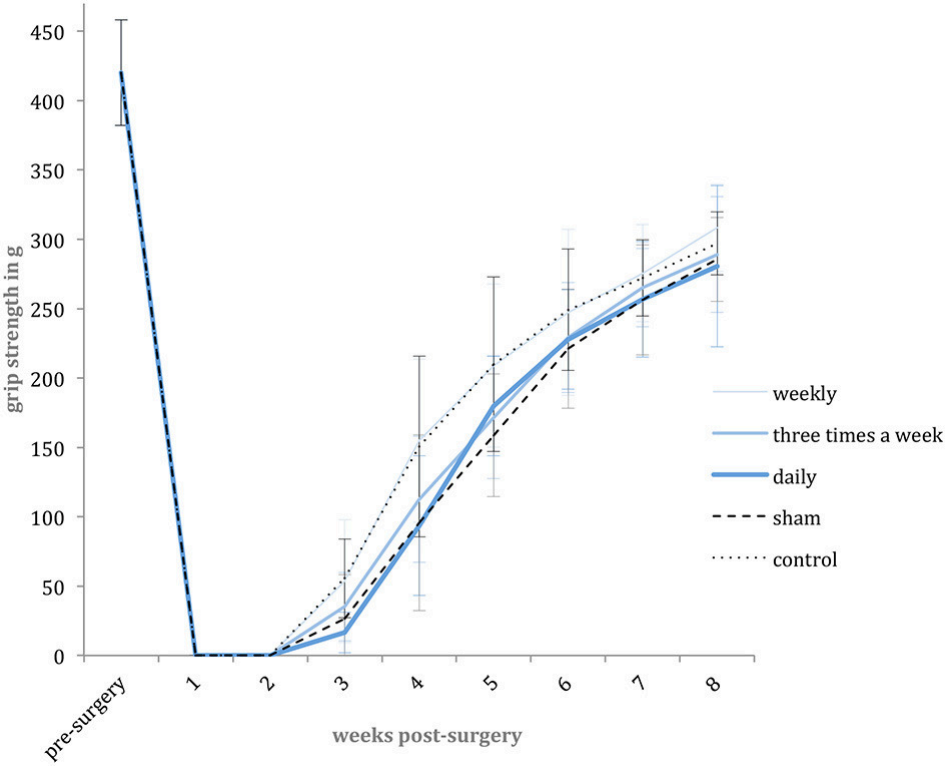
Grip strength level. Shown are the mean pre-surgery grip strength and weekly post-surgery mean grip strength values in gram [g] with standard deviation for each experimental group.

For repetitive assessment of the motor function recovery, weekly grip strength tests were performed during the experimental period. The necessary grip reflex was reliably inducible in all animals. The pre-surgical mean grip strength of the included animals was 442.08 g (SD: 38.05 g; 95% CI: 420.86–463.31 g, *n* = 58). Following surgery, the voluntary finger flexion was initially absent and regenerated later during the experiment. In the first two post-operative weeks, none of the animals showed any active finger flexion, whereas in the third week, recurring grip strength was detected in all groups. From week four to eight, the grip strength increased in all groups without any significant differences among them. During the experimental period, none of the included animals achieved a full recovery of grip strength compared to the pre-operative strength level (Figure 4).

### Electrodiagnostics

Eight weeks post-surgery, electrodiagnostics were successfully performed in all animals.

**Figure 5:**
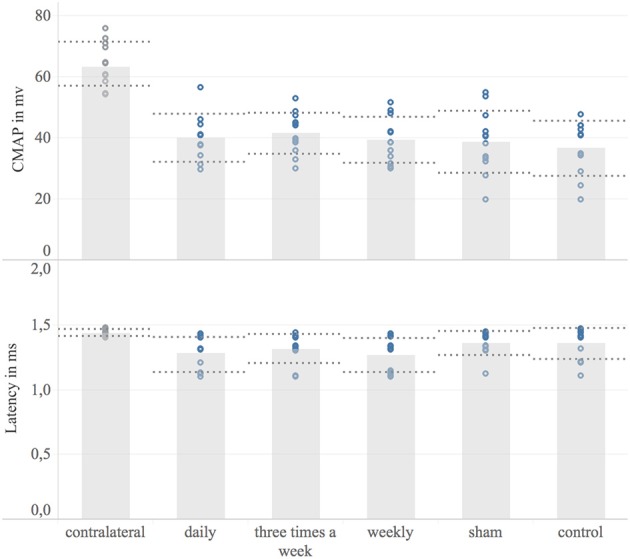
Electrodiagnostic evaluation. Shown are bar graphs of the mean maximum compound muscle action potential (CMAP) in millivolt [mV] **(Upper)** and the distal latency in milliseconds [ms] **(Lower)** with standard deviation (dashed lines) combined with scatter plots of the measured data (dots). Underlying electrodiagnostic analyzes were conducted eight weeks post-surgery.

The non-injured contralateral limb served as a reference, representing the CMAP of an intact neuromuscular unit. Eight weeks following nerve surgery, none of the animals achieved a full recovery of the CMAP compared to the contralateral forelimb (non-injured mean: 63.5 mV; 95% CI: 59.5–67.5 mV, *n* = 12). Comparing the mean operated side CMAP, there was no significant difference between study groups. The corresponding data is shown in Figure 5. Moreover, no significant difference was detected in mean distal latencies among groups. The mean distal latency of the operated nerves did not significantly differ from the values of non-injured, contralateral control.

### Target Muscle Wet Weight

**Figure 6:**
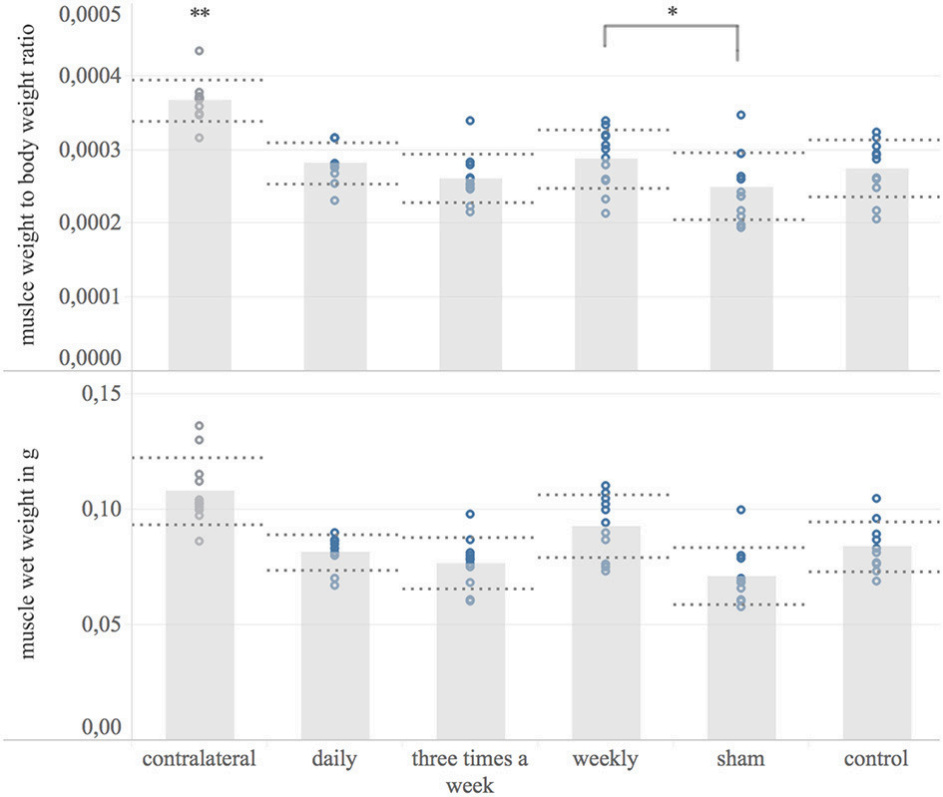
Target muscle wet weight. Shown are bar graphs of the mean muscle wet weight to body weight ratio **(Upper)** and the mean wet weights of the ipsilateral flexor digitorum superficialis muscle in microgram [μg] **(Lower)** with standard deviation (dashed lines) combined with scatter plots of the measured data (dots) 8 weeks post-surgery. ^*^The weekly treated group showed significantly higher muscle weight to body weight ratio compared to the sham-group. ^**^The contralateral muscle weight normalized to body weight was significantly higher compared to the operated side among all experimental groups.

The ipsilateral FDS, as a target muscle of the reconstructed median nerve, was weighted 8 weeks post-surgery to quantify the denervation induced muscular atrophy. The contralateral muscle served as a reference. All groups showed a significant muscular atrophy compared to the contralateral muscle (*p* < 0.01) Figure 6. The weekly treated group (mean: 92 μg, 95% CI: 84–100 μg, *n* = 11) had significantly heavier target muscles and target muscle weight to body weight ratio than the sham group (mean: 71 μg; 95% CI: 63–79 μg, *n* = 12) (*p* = 0.04). No significant difference in target muscle weight to body weight ratio was detected among other groups.

### Histological Analysis

**Figure 7:**
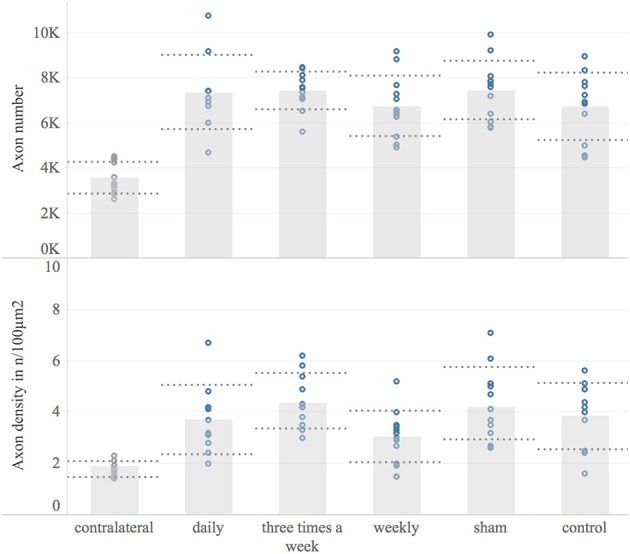
Histological examination. This Figure shows the histomorphological data 8 weeks post-surgery for each experimental group and the contralateral reference. Displayed are bar graphs of the mean myelinated axon number **(Upper)** and the axon distribution density in axons/100 μm^2^ **(Lower)** with standard deviation (dashed lines) and combined with scatter plots of the measured data (dots). Microscopic analyzes were conducted on a constant level 6–8 mm distal to the nerve suture site.

Eight weeks following nerve surgery, the median nerve was harvested and analyzed for myelinated axon number and –distribution density, 6–8 mm distal to the lesion site. The contralateral median nerve served as a reference for the physiological myelinated axon number (mean 3,564; 95% CI: 3,092–4,036; *n* = 12). In all study groups the myelinated axon number distal to the nerve suture was significantly increased compared to the contralateral median nerve (*p* < 0.001) Figure 7. Among study groups no significant difference in mean myelinated axon number of the reconstructed nerve was detected. The myelinated axon distribution density has been found to be significantly increased in the operated nerve compared to the contralateral median nerve in any group (*p* < 0.08), except the weekly treated group, showing a non-significant increase (*p* = 0.24). In accordance with the absolute axon numbers, there was no significant difference in myelinated axon distribution density of the reconstructed nerve among experimental groups.

## Discussion

Despite improved diagnosis and surgical therapy, the limited axonal regeneration following nerve injury leads to persistent disability with a significant burden for patient and society, necessitating new adjunct treatment strategies (1, 2).

In other disciplines the repetitive tissue stimulation via therapeutic ultrasound is already part of the clinical routine (25, 26). Here, Britain's National Institute for Health and Care Excellence (NICE) recommends ultrasound therapy as it offers significant benefits to patients at very low costs (27). Safety analyzes of the NICE Assessment Centre found no reports of any device-related adverse events and identified no significant safety concerns about the recommended ultrasound transducers (27). Our recent meta-analysis of 10 preclinical *in-vivo* studies comprising 445 animals, found repetitive ultrasound therapy with intensities between 200 and 500 mw/cm^2^ to significantly accelerate axonal regeneration and muscle reinnervation, increase the number and myelination of axons distal to the lesion site, and improve nerve conduction velocity after nerve injury in the absence of negative side effects (13). Here, we found the lowest intensity-range of 200–300 mw/cm^2^ to be most efficacious, whereas intensities below 200 mW/cm^2^ have not been investigated following peripheral motor nerve injury yet. Since clinical data for ultrasound stimulation following nerve surgery is currently lacking, the investigation of already available and for other conditions clinically well-tested transducers may facilitate a rapid bench-to-bedside translation. FDA-approved ultrasound therapy devices for bone healing stimulation provide low ultrasound intensities of 30 mW/cm^2^ and 1.5 MHz frequency. Identical transducer parameters have recently been proven to enhance the morphological and functional regeneration of sensory nerves in an experimental nerve injury model (28). However, none of these already clinically available ultrasound transducers has been investigated for peripheral nerve injuries yet. Based on the preexisting evidence, we hypothesized these ultrasound devices to beneficially affect motor nerve regeneration as well and evaluated the FDA-approved EXOGEN bone-healing transducer as adjunct therapy after median nerve injury and primary reconstruction in order to provide a fast clinical translation of this promising preclinical approach.

In previous ultrasound studies, the treatment interval varied widely from daily to once every week, with all showing beneficial effects (13). To identify the most efficacious treatment regimen for the EXOGEN ultrasound device, three different treatment intervals have been chosen for *in-vivo* evaluation, based on the available literature: Daily ultrasound application, 3 times a week and weekly application. To assess the motor function recovery, surrogate estimates such as the sciatic functional index (SFI) were avoided and the isolated target muscle force *in-vivo* was evaluated as an objective measure. Therefore, Bertelli's grasping test was adapted using a custom-made tool, in order to achieve an optimum isolation of the finger flexors, which are known to be exclusively innervated by the reconstructed median nerve (19–21).

These weekly grip strength measurements detected an initially complete palsy of the finger flexors following nerve surgery, and a recurring grip strength in week three for all study groups. This demonstrates the desired complete discontinuity and successful reconstruction of the median nerve in all included animals. These findings further correspond to the nerve conduction recovery assessed via CMAP and distal latency measurements, as well as to the great number of myelinated axons distal to the lesion site as demonstrated in histological analyses. The recovery of function in the third week following surgery indicated the beginning of target muscle reinnervation. However, none of the included animals achieved the pre-surgery grip strength level. These lasting force deficits typically occur following neurotmetic nerve injury even when the nerve is immediately repaired (29–31).

In our previous systematic review an meta-analysis, the motor function recovery was significantly accelerated via repetitive ultrasound application (13). In contrast, none of the EXOGEN treatment groups achieved a significantly superior grip strength compared to sham and control group. Accordingly, histological analyzes of the ultrasound treated and non-treated nerves, found no significant differences regarding myelinated axon number and axon distribution density. This is in contrast to the results of previous works demonstrating an NGF-induced axonal sprouting via ultrasound therapy *in-vivo* and *in-vitro* (11, 13, 32–34). However, whereas the ultrasound frequency, treatment duration and application intervals in our investigation were similar to previous experimental works that demonstrated a significantly improved recovery of ultrasound treated nerves, these studies applied a 6 to 16 fold stronger ultrasound intensity (200 to 500 mW/cm^2^) compared to the evaluated EXOGEN bone-healing device (30 mW/cm^2^). Given the underlying mechanism of ultrasound, the applied sound wave intensity is obviously essential for a therapeutic response. The beneficial effect of ultrasound therapy on nerve regeneration are based on an improved early inflammatory response and accelerated wallerian degeneration, enhanced expression of pivotal neurotrophins such as nerve growth factor (NGF) and ciliary neurotrophic factor (CNTF) as well as an increasing activity and number of supporting Schwann cells (9–12, 22, 35). A direct interaction with cell surface mechano-receptors, converting the mechanical sound wave into biochemical signaling have been found to be involved in such tissue stimulative effects (36, 37). Moreover, the repetitive, high frequency rarefaction and compression of the permeated tissue induce micro-scale turbulences of inter- and intra-cellular fluids. These pressure changes result in rapid formation of micro cavities (38). Close to cell membranes, such cavitation results in surface shear stresses, known to temporarily enhance the cell membrane permeability (39–42). This phenomenon, termed sonoporation, includes increased diffusion rates of transmembrane ion channels as well as macromolecules and consequently affects the target-cell metabolism (43–48). Beside the obvious utilization of cavitation-induced sonoporation in targeted drug delivery it is postulated to be a central mechanism of therapeutic ultrasound in soft-tissue (43, 49–51). Here, the number of cavitation events per unit time is proportional to the applied ultrasound intensity (52). Based on this evidence, we hypothesize the ultrasound intensity provided by the EXOGEN-transducer to be insufficient to generate these required tissue pressure changes. As a result, the cavitation threshold was not exceeded and the associated biological effects were absent. This is in accordance with our electrodiagnostic results. The CMAP and distal latency were not significantly different in treatment groups compared to non-treated groups, contrary to previous studies (22, 23). Since the CMAP represents the summation of individual motor unit action potentials, it corresponds to the number of reinnervated muscle fibers. Here ultrasound-induced axonal regrowth acceleration, as demonstrated for higher intensities, would have led to earlier muscle reinnervation and consequently increased CMAP amplitude. However, none of these effects occurred following ultrasound treatment via EXOGEN bone-healing device. Considering the absence of any treatment effect on functional, histological and electrodiagnostic outcomes, the significant muscle weight differences among weekly treated and sham-group are insufficient to suggest a treatment effect of EXOGEN and may have arisen by chance.

For other conditions such as delayed healing fractures, the EXOGEN therapy is clinically well established and has been proven to be effective in a multi-center randomized sham-controlled trial (14, 26). Here, an extensive body of research investigated the underlying mechanisms for improved bone healing via ultrasound therapy. Yet, nanoscale motion at the fracture site, with consecutively enhanced expression of osteogenic genes and finally up-regulated enchondral ossification and mineralization has been identified as involved mechanisms (53). Given the different biophysical target tissue characteristics and presumed ultrasound mechanisms in peripheral nerve surgery, this offers an explanation for condition-specific ideal treatment parameters such as the different ultrasound intensities.

The tissue stimulative effect of the EXOGEN ultrasound transducer is clinically validated whereas currently no data for experimental rat models exists. Given the significantly different preclinical treatment conditions regarding permeated skin and tissue quality, this represents a potential limitation of the investigated animal model. We addressed this potential limitation by proving a standardized, close-to-clinic application procedure on immobilized limbs, shaved skin and with market-approved ultrasound coupling gel. Moreover, considering the superior regenerative capacity and limited regeneration distance in any rodent nerve injury model, slight treatment effect sizes on axonal regeneration may not become statistically significant in rat.

Finally, the repetitive application of ultrasound with an intensity of 30 mW/cm^2^ had no significant effect on muscle reinnervation or motor function recovery following reconstructive nerve surgery. Moreover, no significant effect has been found for myelinated axon number distal to the lesion site as well as their signal conduction behavior. Consequently, current commercially available and FDA-approved low intensity ultrasound transducers are insufficient to support nerve regeneration. As market-approved ultrasound devices that provide efficacious output-intensities of 200–300 mW/cm^2^ are currently unavailable, transducer modifications are required to extend their clinical scope to nerve surgery.

## Conclusion

Since previous works found ultrasound therapy to significantly improve the functional outcome following nerve surgery, we investigated a clinically well-tested, market-leading bone-healing ultrasound device following primary nerve reconstruction (54). In our study, various regimen of EXOGEN therapy did not beneficially affect the investigated aspects of nerve regeneration. However, conclusive preclinical studies prove ultrasound intensities of 200 to 500 mW/cm^2^ to significantly promote axonal regeneration and accelerate muscle reinnervation following nerve injury. Given the dose-dependent effects in previous works and the underlying biological mechanism of ultrasound therapy, devices with output-intensity of 30 mW/cm^2^ are presumably insufficient to stimulate axonal regeneration. We therefore recommend the clinical investigation of devices providing an ultrasound intensity of 200–300 mW/cm^2^.

## Data Availability Statement

All analyzed datasets for this study are included in the manuscript.

## Author Contributions

PS, LH, SD, UK, and AB: collaborated in the scientific conception and experimental design; PS, SD, and LH: performed the nerve surgeries; SD carried out the experimental sessions, outcome analyses and data acquisition; BH performed the statistical analysis; SD and PS: collaborated in analysis or interpretation of the dataset. All authors contributed their specific expertise during design and completion of the experimental study. SD, KB, and PS: wrote the manuscript; AB, UK, BH, and LH: revised it critically.

### Conflict of Interest Statement

The experimenters received six EXOGEN ultrasound-devices as a temporary, free-of-charge loan from Bioventus LLC. Otherwise, the authors declare that the research was conducted in the absence of any commercial or financial relationships that could be construed as a potential conflict of interest.
